# How and to What Extent Immunological Responses to SARS-CoV-2 Shape Pulmonary Function in COVID-19 Patients

**DOI:** 10.3389/fphys.2021.628288

**Published:** 2021-06-29

**Authors:** Pierantonio Laveneziana, Christian Straus, Silke Meiners

**Affiliations:** ^1^Assistance Publique - Hôpitaux de Paris (AP-HP), Groupe Hospitalier Universitaire APHP-Sorbonne Université, Sites Pitié-Salpêtrière, Saint-Antoine et Tenon, Service des Explorations Fonctionnelles de la Respiration, de l’Exercice et de la Dyspnée (Département R3S), Paris, France; ^2^Sorbonne Université, INSERM, UMRS 1158 Neurophysiologie Respiratoire Expérimentale et Clinique, Paris, France; ^3^Comprehensive Pneumology Center (CPC), University Hospital, Ludwig-Maximilians University, Helmholtz Zentrum München, Member of the German Center for Lung Research (DZL), Munich, Germany

**Keywords:** COVID-19, immune response, respiratory function testing, respiratory physiology, diffusing capacity for lung carbon monoxide

## Abstract

COVID-19 is a disease caused by a new coronavirus SARS-CoV-2, primarily impacting the respiratory system. COVID-19 can result in mild illness or serious disease leading to critical illness and requires admission to ICU due to respiratory failure. There is intense discussion around potential factors predisposing to and protecting from COVID-19. The immune response and the abnormal respiratory function with a focus on respiratory function testing in COVID-19 patients will be at the center of this Perspective article of the Frontiers in Physiology Series on “The Tribute of Physiology for the Understanding of COVID-19 Disease.” We will discuss current advances and provide future directions and present also our perspective in this field.

## Introduction

COVID-19 is a disease caused by a new coronavirus SARS-CoV-2, primarily impacting the respiratory system. COVID-19 can result in mild illness or serious disease leading to critical illness and requires admission to ICU due to respiratory failure.

A major unresolved conundrum is the large spectrum of clinical presentations of patients with COVID-19, ranging from asymptomatic infections or symptomatic mild infections with fever, headache or mild respiratory symptoms (like cough or sore throat) and malaise in 80–85% of patients to flu-like illness and viral pneumonia. Within the “pneumonia phenotype” we also have a large clinical and pathophysiological spectrum that extends from only minor opacification with near normal chest radiographs and mild hypoxemia (in ∼80% of hospitalized patients). Some of these patients develop an acute respiratory failure with severe hypoxemia of quick progression to a phenotype presenting with greater hypoxemia and higher respiratory rates (∼15% of hospitalized patients) to severe diseases manifestations. Seriously ill patients develop severe hypoxemia requiring mechanical ventilation. Their CT scans document edema in the lower lobes, Angio-CTs detect multiple ground-glass opacities often showing micro-embolic lesions and lung ultrasonography that are consistent with interstitial injury with B lines (white lung). This latter phenotype is compatible with an organizing pneumonia with hypoxic vasoconstriction associated with severe hypoxemia (∼2/3 of patients requiring mechanical ventilation). The last phenotype, less common than the previous one, represents an advanced stage with associated acute lung injury requiring mechanical ventilation (Rello et al., 2020). A subset of severe COVID-19 patients also present with coagulation defects with elevated levels of D-dimers and fibrinogen suggesting thrombotic microangiopathy and vasculopathy in the gas-exchange networks and systemically (Huertas et al., 2020; Iba et al., 2020; Vinayagam and Sattu, 2020). This latter phenotype suggests a combination of respiratory and vascular dysfunction in the lungs of severely ill COVID-19 patients which was confirmed in several pathological studies recently (Ackermann et al., 2020; Potus et al., 2020). The particular feature of SARS-CoV2 to induce both respiratory and vascular dysfunction has been established in the past year (Del Turco et al., 2020; Varga et al., 2020).

**FIGURE 1:**
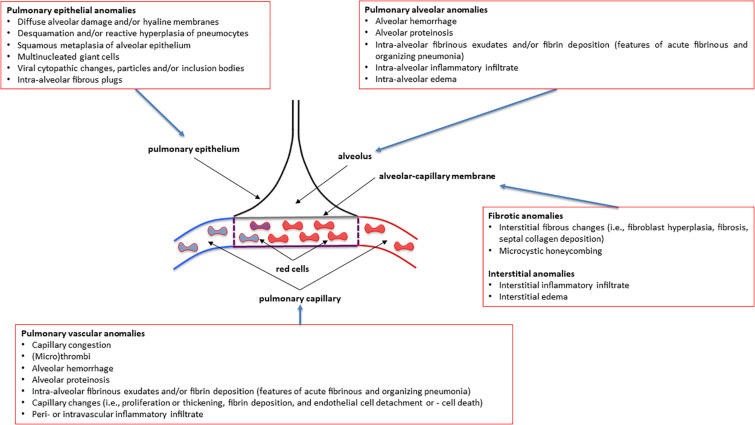
Overview of the most common pulmonary pathology findings observed in post-mortem patients affected by various degrees of severity of COVID-19 (coronavirus disease 2019). See the text for more details and explanations. This is an original figure, no permission to reproduce is required.

Increasing evidence suggests that these diverse clinical phenotypes might be explained by the immunological failure to control and restrict SARS-CoV2 infection of the lung. Failure and skewing of the adaptive immune system, promiscuous infection of epithelial (pneumocytes), endothelial as well as immune cells, coagulation defects and uncontrolled neutrophilic activation potentially govern the impact of COVID-19 on respiratory function and clinical phenotypes (Ackermann et al., 2020; Casadevall and Pirofski, 2020; Del Turco et al., 2020; Gordon et al., 2020a; Tay et al., 2020; Figure 1). An increased understanding of the immunological dysfunction underlying the different clinical phenotypes of COVID-19 survivors impacts the management of clinical and pathophysiological consequences of this disease. The immune response and the abnormal respiratory function with a focus on respiratory function testing in COVID-19 patients will be at the heart of this Perspective article of the Frontiers in Physiology Series on “The Tribute of Physiology for the Understanding of COVID-19 Disease.” We will discuss current advances and provide future directions and present also our perspective in this field.

## Respiratory Immunology

SARS-CoV-2 infection primarily targets the respiratory tract. For the virus to effectively enter the host cell it requires the membrane expression of angiotensin-converting enzyme-2 (ACE2) together with cofactors, such as transmembrane serine protease 2 (TMPRSS2), and furin (Hoffmann et al., 2020; Lukassen et al., 2020; Sungnak et al., 2020; Wang et al., 2020). A plethora of studies analyzed expression of these factors in lung cells using single cell RNA sequencing (scRNA seq), *in situ* hybridization as well as immunohistochemistry profiling (Hikmet et al., 2020; Hou et al., 2020; Lukassen et al., 2020; Sungnak et al., 2020; Ziegler et al., 2020). Despite some surprising controversies, the emerging view is that ciliated airway cells and alveolar type 2 epithelial cells are the primary targets for SARS-CoV-2.

Upon virus infection, cells directly respond to the infectious agents by activating a protective type I interferon program. This includes the secretion of type I interferons (IFN I) that serve to initiate, amplify, and/or sustain host inflammatory responses (Martin et al., 1997; Yoshikawa et al., 2009). In particular, monocytes/macrophages and natural killer (NK) cells are IFN-responsive cells of the innate immune system. They play a major role in raising an efficient T cell-mediated adaptive immune response and thereby determine the outcome of virus infections (Frieman et al., 2008; Lazear et al., 2019). NK cells directly kill virally infected cells via their spontaneous cytolytic activity and activate the innate and adaptive immune system via secreting a variety of soluble mediators. Monocytes have specialized roles in the human lung (Landsman et al., 2007; Bassler et al., 2019; Kulikauskaite and Wack, 2020). Tissue resident macrophages, such as alveolar macrophages (AM) of the lung play a major role in early innate immunity against infections and environmental challenges (Kopf et al., 2015; Wynn and Vannella, 2016; Watanabe et al., 2019). Together with patrolling monocytes these macrophages engulf apoptotic cells and can cross-present engulfed antigen to T cells eliciting adaptive immune responses (Kopf et al., 2015; Thomas et al., 2015). Activation of cytotoxic CD8^+^ T cell responses is essential for fast clearing of infected cells and maintaining long-term suppression of viral infections via immunological memory (Zuniga et al., 2015; Schmidt and Varga, 2018). This type I interferon triggered immune activation allows confinement of the virus infection in a time (usually within 3 days) and spatial manner and development of adaptive immune cell memory (Schmidt and Varga, 2018).

SARS-CoV2 effectively suppresses type I interferon responses in infected cells. Several SARS-CoV and SARS-CoV2 encoded viral proteins have been demonstrated to interfere with the IFN I signaling pathway (Kopecky-Bromberg et al., 2007; Shi et al., 2014, 2019; Gordon et al., 2020b)^1^. The infected cells of the respiratory epithelium thus fail to launch a robust IFN-I response to SARS-CoV2 but at the same produce exuberant inflammatory cytokines which disrupts a balanced anti-viral innate and adaptive immunity (Angka et al., 2020; Blanco-Melo et al., 2020; Schultze and Aschenbrenner, 2021). Failure of the adaptive immune system to confine the SARS-CoV2 infection to the upper respiratory tract within the first days post infection may contribute to the observed biphasic disease course in patients that develop COVID-19 pneumonia (Jesenak et al., 2020; Tay et al., 2020). Indeed, in the blood, the number of adaptive immune cells and their functionality is reduced in severely ill patients (Zhou et al., 2020). In particular, lymphopenia is the most consistent laboratory abnormality in severe COVID-19–infected patients. Progressive lymphodepletion and signs of T cell exhaustion are observed in patients who clinically deteriorate with severe COVID-19 (Diao et al., 2020; Zhang et al., 2020). In contrast, reappearance of effector T cells associates with recovery from the disease (Odak et al., 2020). Moreover, lungs from patients who succumbed to SARS-CoV2 show extensive cellular immune infiltrates with macrophages representing a prominent cell type (Barton et al., 2020; Ruscitti et al., 2020; Tian et al., 2020b; Schultze and Aschenbrenner, 2021). These data strongly indicate that a defective type I interferon response at the onset of infection is key to the observed uncontrolled immune responses, such as excessive cytokine production (”cytokine storm”) and impaired protective T cell responses (Gordon et al., 2020b; Tay et al., 2020). This scenario might involve an imbalanced activation of NK and T cells as described for severe influenza infections (Frank and Paust, 2020).

Defective local and temporal confinement of SARS-CoV2 virus replication due to impaired type I interferon responses in the primarily infected lung epithelial cells may result in a spill-over of the infection into the vascular system of the lung. Such spreading of the infection will be fostered by excessive inflammatory signaling in the alveolus that causes disruption of the basement membrane, leakiness of alveolar capillaries and massive immune cell recruitment. SARS-CoV2 is able to infect endothelial and immune cells (Ackermann et al., 2020; Huertas et al., 2020; Iba et al., 2020; Potus et al., 2020)^2^ suggesting some promiscuity in terms of host cell selectivity (tropism) as suggested previously as a general feature of Coronoviruses (Hulswit et al., 2016). The tropism of a virus is defined as its ability to infect specific cell types, organs or species. This capacity depends on the expression of receptors for viral entry, e.g., ACE2 for SARS-CoV2, and cofactors, such as TMPRSS2 and furin, but also on the permissiveness of the cell to allow virus replication and support productive infection. Accordingly, viral tropism is determined by multiple viral and host cell factors (Adler et al., 2017). Not only more and more host factors are identified that fine-tune SARS-CoV2 entry and replication in host cells (Hou et al., 2020), but also new receptors are being uncovered, such as for example neuropilin-1, which is highly expressed in fibroblasts, brain and endothelial cells (Cantuti-Castelvetri et al., 2020)^3^. These factors might determine the rather broad tropism of SARS-CoV2 as observed in autopsied lungs of COVID-19 patients (Ackermann et al., 2020; Huertas et al., 2020; Iba et al., 2020; Potus et al., 2020) and in *ex vivo* models and cells (Hui et al., 2020). Promiscuity of the SARS-CoV2 with regard to host cell tropism together with unconfined infection resulting in high local virus load might then result in aberrant infection of endothelial and recruited immune cells in addition to inflammatory activation of these cells.

Damaged respiratory epithelial cells and pulmonary endothelial dysfunction activate platelets and formation of intravascular microthrombi (Connors and Levy, 2020; Del Turco et al., 2020). These pathophysiological changes will contribute to impaired hypoxemic vasoconstriction and the clinical phenotype of “happy hypoxemia” in COVID-19 patients (Dhont et al., 2021). If the immune system doesn’t gain control, endothelial dysfunction and coagulation defects might spread systemically causing vasculitis, disseminated intravascular coagulopathy (DIC) and immunothrombosis (Engelmann and Massberg, 2013; Jackson et al., 2019). This scenario is fully supported by the recent study on SARS-CoV2 infected Macaques that developed severe vascular disease and pulmonary thrombosis (Aid et al., 2020). Defective coagulation has been observed in severe COVID-19 patients as evidenced by thrombocytopenia, elevated levels of D-Dimer and of fibrin/fibrinogen degradation products (for an overview see Iba et al., 2020).

How can we relate the above-described immune dysregulation in severely ill COVID-19 patients to altered lung pathology and respiratory function? As the presence of D-Dimer correlates with reduced lung function and DLCO, one could envision that coagulation defects in the lung capillaries and the formation of microthrombi contribute to impaired gas exchange in COVID-19 patients with severe disease (Figure 1; Zhao et al., 2020). Gas exchange will also be hampered by extracellular matrix deposition upon repair of the damaged respiratory epithelium in COVID-19 survivors (Figure 1). Fibrotic remodeling of the lung might be driven by skewing of adaptive T cell responses toward impaired regulatory T cell (CD4^+^ Treg) function and increased Th17 differentiation as observed in severely ill COVID-19 patients (De Biasi et al., 2020; Zhang and Zhang, 2020). Th17 is a well-known CD4^+^ T cell subset causally involved in organ fibrosis including the lung (Barron and Wynn, 2011; Way et al., 2013). Along these lines, reduced numbers of CD4^+^ Tregs have been observed in severe cases of COVID-19 together with increased levels of cytotoxic follicular helper cells and cytotoxic T helper cells (Meckiff et al., 2020). An additional line of evidence suggests the involvement of inflammatory neutrophils that persist in the lung. Recent data from autopsies of deceased COVID-19 patients demonstrated prominent activation of neutrophils in the lung with extracellular NET formation and alterations in extracellular matrix deposition together with multiorgan dysfunction (Schurink et al., 2020; Wu et al., 2020). Of note, in some patients the viral infection load of the diseased lung tissue was minimal suggesting that the immune system was unleashed at some point independent of the virus infection (Casadevall and Pirofski, 2020; Wu et al., 2020). An unpublished preprint suggests that neutrophil NET formation promotes the transition of lung epithelial cells toward a mesenchymal phenotype that might contribute to fibrotic lung remodeling^4^.

## Physiology and Pathophysiology of Abnormal Pulmonary Function Variables as Observed in COVID-19 Survivors

Altered lung diffusion capacity is the most common anomaly followed by restrictive ventilatory defect. This section attempts to describe the physiology and pathophysiology that underlies the three most common abnormal pulmonary function variables observed in COVID-19 survivors: TLCO, TLCO/VA and Total Lung Capacity. A particular focus will be paid on highlighting the difference between TLCO and TLCO/VA and on what is important about having a greater decline in TLCO than in TLCO/VA, and how this feeds back to lung pathology.

**FIGURE 2:**
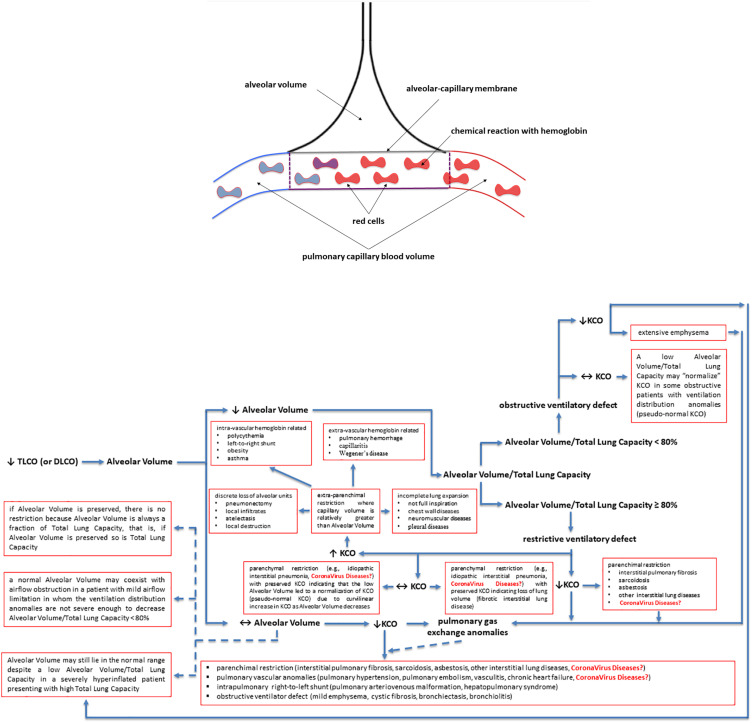
Factors contributing to a decreased lung transfer (or diffusing) capacity for carbon monoxide (CO) (TLCO or DLCO) and the algorithm that allows physiologists and clinicians to unravel its mechanisms. If TLCO (or DLCO) is reduced, the next step is to check whether the Alveolar Volume is preserved or reduced. If Alveolar Volume is diminished, the next step is to check whether the Alveolar Volume/Total Lung Capacity ratio is low (<80%) due to ventilation maldistribution secondary to an obstructive ventilatory defect or is preserved (≥80%) due to restrictive ventilatory defect, associated or not with impaired pulmonary gas exchange. If Alveolar Volume is preserved, please follow the arrows in the algorithm to get some explanations and to see whether the KCO is reduced and there are pulmonary gas exchange anomalies associated with. KCO, transfer or diffusion coefficient (KCO = TLCO/Alveolar Volume or DLCO/Alveolar Volume); Coronavirus diseases is written in red as potential yet not fully understood mechanisms explaining the TLCO or DLCO anomalies observed in Coronavirus diseases, such as COVID-19 (caused by SARS-CoV-2), SARS (caused by SARS-CoV-1) and MERS (caused by MERS-CoV); SARS, Severe Acute Respiratory Syndrome; MERS, Middle East Respiratory Syndrome; CoV, coronavirus; COVID-19, coronavirus disease 2019. See the text for more details and explanations. This is an original figure, no permission to reproduce is required.

The lung transfer (or diffusing) capacity for carbon monoxide (CO) [TLCO or DLCO; TLCO being more commonly used in North-America whereas DLCO being more commonly used in Europe] reflects the capacity of CO transfer from the environment to the pulmonary capillary blood and represents the most clinically practical standard methodology to assess the gas exchange in the lung. In this review we will use the term TLCO. KCO, the transfer or diffusion coefficient is the rate constant for CO uptake from alveolar gas and is impacted mostly by the thickness and area of the alveolar capillary membrane, the volume of blood circulating in pulmonary capillaries coupling ventilated alveoli and the concentration and properties of hemoglobin in the alveolar capillaries blood (Figures 2A,B). KCO and the alveolar volume (VA) are the two main factors that determine TLCO (Figures 2A,B). From a mathematical standpoint, KCO can be calculated as TLCO/VA under BTPS conditions (Body Temperature, ambient Pressure, Saturated with water vapor). It should be noted that TLCO/VA is not a simple ratio as the relationship between lung volume and CO uptake is certainly less than 1:1 (Hughes and Pride, 2012). The use of KCO has recently been recommended instead of TLCO/VA, as TLCO/VA may be interpreted that TLCO can be normalized for VA (Graham et al., 2017).

A low TLCO is not exclusively determined by reduced VA (Nusair, 2020), and residual interstitial anomalies (Chen et al., 2020; Mo et al., 2020; Qin et al., 2021) and pulmonary vascular anomalies (i.e., abnormal capillary-alveolar units) (Morris et al., 2021) may play a fundamental role and this could be also the case in COVID-19 survivors (Figures 2A,B). This holds true as the interpretation of low TLCO must consider the complex relationship between VA, TLCO and KCO, and may inopportunely exclude the presence of abnormal gas exchange in the lung (Figures 2A,B). To prove this point, we can use data from “severe pneumonia” COVID-19 related patients discussed in this review to model according to Hughes and Pride (2012) what TLCO and KCO responses would be expected if VA was diminished as a consequence of either suboptimal alveolar expansion or due to loss of alveolar units while having a normal expansion in communicating alveoli. We would then observe two trajectories: the first one is that the decline in TLCO would be largely greater than expected if a decrease in VA was the unique anomaly, regardless of the mechanism behind the diminished VA; the second one is that a decrease in VA due to either above-mentioned mechanism would be associated with an augmentation in KCO, which would be contrary to the diminished KCO observed in many of the discharged patients with severe COVID-19. Therefore, the decrease in KCO may suggest that loss of alveolar units is not sufficient to determine the observed alteration in TLCO. Thus, while the anomalies in TLCO observed in “severe pneumonia” COVID-19 related patients explored in several studies may be partially explained by diminished VA, the decrease in KCO measured together with the diminished VA also implies that abnormal gas exchange in the lung occurs. Now, the question arises as whether this is due to anomaly of the alveolar-capillary barrier or to abnormal pulmonary blood volume. Unfortunately, this cannot be easily determined based on data presented in these studies. Lung fibrosis associated with acute respiratory distress syndrome in COVID-19 patients, would likely alter alveolar-capillary units, giving rise to loss of alveolar units and altered gas exchange in the lung. The consequence would be a decrease in both VA and KCO (for that diminished VA). There is mounting evidence for impaired pulmonary hemodynamics in COVID-19 patients (Potus et al., 2020), including vascular pruning, decreased pulmonary blood volume and abnormal pulmonary blood volume distribution as measured via high resolution CT (Lins et al., 2020; Morris et al., 2021). Figures 2A,B shows that a decrease in KCO may develop in the context of alveolar-capillary damage, microvascular pathology, or anemia. Factors responsible for a reduced VA are numerous and may include decreased alveolar expansion, alveolar damage or loss, or inspired gas maldistribution in the context of obstructive ventilator defect. Therefore, when KCO turns normal, in the presence of a low TLCO, it is associated with reduced VA, thus indicating a restrictive ventilator defect (see below and Figures 2A,B). This is because only the functional alveolar units have been sampled thereby providing an erroneous picture toward more preserved areas of the lungs (Figures 2A,B). It should be noted that if VA is preserved, there is no restrictive ventilatory defect because VA is always a fraction of Total Lung Capacity, i.e., if VA is preserved so is Total Lung Capacity (Figures 2A,B). To conclude and for the sake of clarity: the same TLCO may occur with various combinations of VA and KCO, each suggesting different abnormal respiratory conditions. It is difficult to interpret which one plays the predominant role because both diminished alveolar volume and KCO concur to the pathogenesis of altered lung diffusion capacity. TLCO gives a global evaluation of gas exchange in the lung, while the alveolar-capillary membrane diffusing capacity only depends on molecular diffusion of the membranes. We would thus need more refined techniques capable of measuring more specifically the alveolar-capillary membrane. These could include measurement of TLCO with inhaled gas mixtures containing two or three different oxygen fractions, or combined TLCO and diffusing capacity measurements of the lung for nitric oxide (DLNO). Such sophisticated analysis would shed light on the precise mechanisms of reduced TLCO in COVID-19 survivors and allows distinguishing between interstitial and pulmonary capillary anomalies (see “Future directions, perspectives and conclusions” section).

The second most common abnormality in COVID-19 survivors is a restrictive ventilatory defect. A restrictive ventilatory defect is defined by a pathologically decreased total lung capacity. If caused by parenchymal lung disease, restrictive ventilator defect is accompanied by reduced gas transfer, which may be marked clinically by desaturation after exercise or even at rest (see the above paragraph).

Total lung capacity is the greatest volume of gas in the lungs achieved after maximal voluntary inspiration. It depends on the static balance between the outward forces generated by inspiratory muscles during a maximal inspiratory effort and the inward elastic forces of the chest wall and lung. It is the lung that normally contributes the most to the elastic recoil forces of the respiratory system at total lung capacity. At total lung capacity, these two sets of forces are equal and opposite in sign. The decrease in total lung capacity usually reflects the reduced lung volumes either because of an alteration in lung parenchyma or because of a disease of the pleura, chest wall, or neuromuscular apparatus that may affect the pressure-generating capacity of the inspiratory muscles or the compliance of the lung or the compliance of the chest wall. Interstitial lung anomalies as such those observed in some forms of COVID-19 (Polak et al., 2020) may result in a restrictive ventilatory defect (Figures 1, 2).

## Abnormal Respiratory Function in COVID-19 Patients

Respiratory function testing has been performed in COVID-19 survivors at the time of hospital discharge and weeks after hospital discharge. This seems an important issue when dealing with COVID-19 survivors as these respiratory function testing anomalies may have a huge impact on the management, independency and quality of life of these patients as well as on the healthcare systems.

### At the Time of Hospital Discharge

In the Fumagalli’ study 13 patients with COVID-19 pneumonia were enrolled and the authors found that at the time of clinical recovery, 10 out of 13 patients presented with a restrictive pattern measured at spirometry: forced expiratory volume in the first second (FEV_1_) and forced vital capacity (FVC) were lower compared to lower limit of normality values, while FEV_1_/FVC was higher compared to the upper limit of normality values. These results obtained in a very small sample size should be taken with caution as measure of Total Lung Capacity, preferably with plethysmography, was not included and the diagnosis of restrictive pattern was made exclusively on the reduced FVC, which is questionable and not acceptable (Pellegrino, 2005). In addition, TLCO measurement was not employed; this would have permitted a better understanding of the origin and the quality of pulmonary gas exchange damage.

In the Mo’ study 110 patients with COVID-19 infection were enrolled, which included 24 cases of mild illness, 67 cases of pneumonia and 19 cases of severe pneumonia (Mo et al., 2020). Spirometry, plethysmography and TLCO tests were performed on the day of or one day before hospital discharge. The authors found that 47% of their patients had anomalies in TLCO, 25% in TLC, 14% in FEV_1_, 9% in FVC, 4.5% in the FEV_1_/FVC ratio and 7% in small airway function. The most interesting observation was the significant difference in impaired TLCO among the different groups of severity, which accounted for 30% in mild illness, 42% in pneumonia and 84% in severe pneumonia, respectively (*p* < 0.05). This trend of the gradual decrease in level of TLCO among patients was identical with the varying degree of severity. Of note, in 50% of the TLCO -impaired patients, the TLCO corrected for alveolar volume (TLCO/VA) was still within the normal range, which might indicate that TLCO decrease was more than the TLCO/VA in recovered subjects. In addition, the value of TLC as% of predicted in severe pneumonia cases was much less than that of pneumonia or mild illness, suggesting higher impairment of lung volume in severe cases. No significant difference among the discharged survivors with different severity in regard to other ventilatory defects (e.g., reduced FEV_1_/FVC) was observed.

These two studies, strongly suggest that respiratory function needs to be carefully investigated in COVID-19 patients, as it was already done for other atypical pneumonia, such as severe influenza A (H1N1) pneumonia (Hsieh et al., 2018). This is because the lung is the most affected organ by COVID-19 and previous other atypical pneumonia, with anomalies that include diffuse alveolar epithelium destruction, capillary damage/bleeding, hyaline membrane formation, alveolar septal fibrous proliferation, and pulmonary consolidation.

### In Discharged Patients

In the same study by Fumagalli and co-workers FVC was still lower than the lower limit of normality after 6 weeks from hospital discharge (Fumagalli et al., 2020). Again here, these results obtained in a very small sample size should be taken with caution as measure of TLC was not included and the diagnosis of restrictive pattern was made exclusively on the reduced FVC, which is questionable and not acceptable (Pellegrino, 2005). Another study by Huang et al. (2020) performed respiratory function testings in 57 COVID-19 patients after 30 days of hospital discharged and found anomalies in 75% of them; 10, 9, 44, 12, and 53% of enrolled patients had FVC, FEV_1_, FEV_1_/FVC ratio, TLC, and TLCO values less than 80% of predicted values, respectively, whereas 49 and 23% of patients presented with maximum static inspiratory and expiratory pressure (PImax and PEmax, respectively) values less than 80% of the corresponding predicted values. Compared with non-severe cases (n = 40), severe patients (n = 17) showed higher incidence of TLCO impairment (76 vs. 43%, p = 0.019), and significantly lower percentage of predicted TLC. Of note, only 11% of patients showed obstructive and 12% restrictive ventilatory defects (Huang et al., 2020). What is also striking yet surprising is that a small percentage of patients with no residual imaging abnormalities presented with a slight decrease in TLCO. Similar to this study, Frija-Masson et al. (2020) observed abnormal lung function in more than 50% of COVID-19 patients after 30 days of hospital discharge. Almost one third of these patients had decreased TLCO values indicating that these patients have lung vascular damage which coincides with data from Huang et al. (Huang et al., 2020).

On the contrary, Rogliani et al. (2020) have recently pointed out that hospitalized patients with mild-to-moderate forms of COVID-19 are not at risk of developing pulmonary fibrosis. In their study, patients were enrolled within two months from hospital discharged and authors found that FEV1 and FVC, both expressed as % predicted, were in the normal range. Here again, these results should be taken with caution as neither measurement of TLC nor of TLCO was included in the study.

Few studies have explored pulmonary function in COVID-19 survivors at 3 (Cortés-Telles et al., 2021; Qin et al., 2021) and 4 (Anastasio et al., 2021) months after hospital discharge. All these studies showed alteration in TLCO (in more than 50% of patients), in total lung capacity (in more than 10% of patients), in pressure generating capacity of respiratory muscles (in less than 40–50% of patients) but to a much lesser extent alterations in the airway functions (in less than 10% of patients). The results of these studies converged to the conclusion that the worst the lung involvement during SARS-COV-2 infection (in those patients who developed acute respiratory distress syndrome or those who required invasive mechanical ventilation) the worst the impairment in pulmonary function after 3–4 months especially in terms of TLCO and the less the likelihood to improve pulmonary function over time. Accordingly, respiratory rehabilitation and gradual physical activity immediately after hospital-discharge should be encouraged as it can slow down or improve respiratory function, such as total lung capacity and TLCO, quality of life and anxiety in these fragile patients (Liu et al., 2020).

In conclusion, several mechanisms, sequential or not, may occur and explain the damages induced by SARS-CoV2 infections of the lungs. They include the microvascular damages with interstitial thickening with clear lungs on radiology exams along with a severe hypoxemia (McGonagle et al., 2020; Tian et al., 2020a), the development of alveolar injury inducing a gradual loss of the alveolar spaces (Tian et al., 2020a), and last but not least the diminished alveolar volume that may be explained by changes in mechanical properties of the lungs and the chest wall and by dysfunction of respiratory muscles after critical illness. These anomalies can be temporary or responsible for a potential long lasting pulmonary parenchymal dysfunction post-COVID-19 (Spagnolo et al., 2020). Given these interplays, two hypotheses on reduced TLCO can be proposed in COVID-19 survivors: (1) a reduced TLCO with normal TLCO/VA may be in favor of definitive alveolar loss/destruction, with no optimistic perspectives of recovering; (2) a reduced TLCO with diminished LCO/VA may be in favor of alveolar lesions (pulmonary capillary and/or membrane anomalies) that are still evolving, with the optimistic perspective of some and at least partial recovery. We should therefore follow-up COVID-19 survivors to see whether they are able to recover from their DLCO anomalies. Few studies have explored some “predictors” for lung function decline, especially for TLCO. Pulmonary interstitial damage (inferred to by the Chest CT total severity score), the development of acute respiratory distress syndrome, and vascular damage (inferred to by the high D-dimer levels at the time of hospital admission) have been pointed out as potential predictors for lung function decline, especially for TLCO but also for TLC (Morris et al., 2021; Qin et al., 2021).

## Specific Features of Respiratory Dysfunction in COVID-19 Compared to Other Viral Pneumonia (Sars, Mers, and Influenza a H1N1)

The observations on anomalies in respiratory function, especially in DLCO, in more than 50% of the COVID-19 survivors raise the question of a potential progression toward lung fibrosis in some patients. Interestingly, the greater decline in TLCO compared to TLCO/VA suggests that impaired diffusion across the membrane may be more causative for pulmonary dysfunction than reduced lung volume. Previous studies have demonstrated that patients that recovered from coronavirus pneumonia still have damaged lungs. Impaired lung function was common and lasted for months or even years. In follow-up studies on rehabilitating SARS patients lasting from half a year to 3 years, impaired TLCO was the most common anomaly, ranging from 15 to 44%, followed by reduced TLC, ranging from 5 to 11% (Hui et al.,2005a,b; Ngai et al., 2010). Park et al. (2018) showed that 37% of MERS survivors still presented with an impairment of TLCO, but normal TLC at 12 months. In addition, pulmonary function improved significantly in the first 3 months but with no further significant improvement from 3 to 6 months after discharge among survivors to severe influenza A (H1N1) pneumonia (Hsieh et al., 2018). Some other studies showed a complete normalization of pulmonary function 6 months after H1N1-related ARDS (Toufen et al., 2011). On the contrary, about 80% of survivors to ARDS not provoked by influenza A H1N1 had reduced diffusing capacity, 20% had airway obstruction, and 20% had restrictive pattern 12 months after recovery (Orme et al., 2003). These data are discordant with preliminary follow-up results on COVID-19 survivors highlighting the greater and persistent decline of pulmonary function (TLCO and total lung capacity) in COVID-19 survivors compared with SARS, MERS, and influenza A (H1N1) survivors.

Studies on lung function in COVID-19 survivors at 6 and 12 months from hospital discharge are thus urgently needed in order to monitor the long-term effect of COVID-19 infection on the respiratory system in patients with severe-to-extremely-severe pneumonia. A prediction would be that at least at 6 months from hospital discharge these patients may still present with an abnormal TLCO and, to lesser extent, a restrictive ventilatory defect.

## Future Directions, Perspectives, and Conclusion

Immunological understanding of early as well as chronic immune responses might be helpful for future stratification of surviving COVID-19 patients with chronic respiratory impairment. In our opinion, potential future directions and perspectives are as follows:

• Pathological and lung function evidence for a vascular component of severe COVID-19 patients which has long-lasting consequences should be explored.
• Immunological evidence on deranged adaptive immune function that may drive fibrotic lung diseases and evidence for impaired diffusion capacity in survivors of severe COVID-19 needs to be evaluated.
• More attention should be paid to COVID-19 survivors presenting with impaired (minor or not) diffusion capacity and perhaps with persistent dyspnea but with no other associated anomalies in chest or CT scan imaging. Techniques capable of measuring more specifically the alveolar-capillary membrane, such as measurement of TLCO including inhaled gas mixtures containing two or three different oxygen fractions or combined TLCO and diffusing capacities of the lung for nitric oxide (DLNO) measurements, are welcome to shed light on the precise mechanisms of reduced TLCO in COVID-19 survivors particularly in distinguishing between interstitial and pulmonary capillary anomalies.
• More particularly, two hypotheses on reduced TLCO could be tested in COVID-19 survivors: (1) a reduced TLCO with normal TLCO/VA may be in favor of definitive alveolar loss/destruction, with no optimistic perspectives of recovering; (2) a reduced TLCO with diminished TLCO/VA may be in favor of alveolar lesions (pulmonary capillary and/or membrane anomalies) that are still evolving, with potential and optimistic perspective of some recovering, at least partial. We should therefore follow-up COVID-19 survivors to see whether they are able to recover from their DLCO anomalies.
• A long-lasting follow-up in terms of respiratory function testing is proposed for COVID-19 survivors as results from literature are conflicting as to whether these patients may fully recover or even develop pulmonary sequelae.

This combined perspective on basic immunological responses and physiological abnormalities might foster a better understanding of the disease course and may also shape future stratification of patients and treatment options.

## Data Availability Statement

The original contributions presented in the study are included in the article/supplementary material, further inquiries can be directed to the corresponding author/s.

## Author Contributions

All authors listed have made a substantial, direct and intellectual contribution to the work, and approved it for publication.

## Conflict of Interest

The authors declare that the research was conducted in the absence of any commercial or financial relationships that could be construed as a potential conflict of interest.

## Footnotes

1 https://www.biorxiv.org/content/10.1101/2020.06.17.156455v1

2 https://doi.org/10.1101/2020.09.25.20195818

3 www.proteinatlas.org

4 https://doi.org/10.1101/2020.11.09.374769

## References

[B1] Ackermann M. Verleden S. E. Kuehnel M. Haverich A. Welte T. Laenger F. (2020). Pulmonary vascular endothelialitis, thrombosis, and angiogenesis in Covid-19. *N. Engl. J. Med.* 383 120–128. 10.1056/nejmoa2015432 32437596 PMC7412750

[B2] Adler B. Sattler C. Adler H. (2017). Herpesviruses and their host cells: a successful liaison. *Trends Microbiol.* 25 229–241. 10.1016/j.tim.2016.11.009 27956142

[B3] Aid M. Busman-Sahay K. Vidal S. J. Maliga Z. Bondoc S. Starke C. (2020). Vascular disease and thrombosis in SARS-CoV-2-infected rhesus macaques. *Cell* 183 1354–1366.e13. 10.1016/j.cell.2020.10.005 33065030 PMC7546181

[B4] Anastasio F. Barbuto S. Scarnecchia E. Cosma P. Fugagnoli A. Rossi G. (2021). Medium-term impact of COVID-19 on pulmonary function, functional capacity and quality of life. *Eur. Respir. J.* 2004015. 10.1183/13993003.04015-2020 33574080 PMC7877327

[B5] Angka L. Market M. Ardolino M. Auer R. C. (2020). Is innate immunity our best weapon for flattening the curve? *J. Clin. Invest.* 130 3954–3956. 10.1172/jci140530 32510470 PMC7410037

[B6] Barron L. Wynn T. A. (2011). Fibrosis is regulated by Th2 and Th17 responses and by dynamic interactions between fibroblasts and macrophages. *Am. J. Physiol. Gastrointest. Liver Physiol.* 300 G723–G728. 10.1152/ajpgi.00414.2010 21292997 PMC3302189

[B7] Barton L. M. Duval E. J. Stroberg E. Ghosh S. Mukhopadhyay S. (2020). COVID-19 autopsies, Oklahoma, USA. *Am. J. Clin. Pathol.* 153 725–733. 10.1093/ajcp/aqaa062 32275742 PMC7184436

[B8] Bassler K. Schulte-Schrepping J. Warnat-Herresthal S. Aschenbrenner A. C. Schultze J. L. (2019). The myeloid cell compartment—cell by cell. *Annu. Rev. Immunol.* 37 269–293. 10.1146/annurev-immunol-042718-041728 30649988

[B9] Blanco-Melo D. Nilsson-Payant B. E. Liu W.-C. C. Uhl S. Hoagland D. Møller R. (2020). Imbalanced host response to SARS-CoV-2 drives development of COVID-19. *Cell* 181 1036–1045.e9. 10.1016/j.cell.2020.04.026 32416070 PMC7227586

[B10] Cantuti-Castelvetri L. Ojha R. Pedro L. D. Djannatian M. Franz J. Kuivanen S. (2020). Neuropilin-1 facilitates SARS-CoV-2 cell entry and infectivity. *Science* 370 856–860. 10.1126/science.abd2985 33082293 PMC7857391

[B11] Casadevall A. Pirofski L. A. (2020). In fatal COVID-19, the immune response can control the virus but kill the patient. *Proc. Natl. Acad. Sci. U.S.A.* 117 30009–30011. 10.1073/pnas.2021128117 33177233 PMC7720099

[B12] Chen R. Gao Y. Chen M. Jian W. Lei C. Zheng J. (2020). Impaired pulmonary function in discharged patients with COVID-19: more work ahead. *Eur. Respir. J.* 56:2002194. 10.1183/13993003.02194-2020 32586883 PMC7315814

[B13] Connors J. M. Levy J. H. (2020). COVID-19 and its implications for thrombosis and anticoagulation. *Blood* 135 2033–2040. 10.1182/BLOOD.2020006000 32339221 PMC7273827

[B14] Cortés-Telles A. López-Romero S. Figueroa-Hurtado E. Pou-Aguilar Y. N. Wong A. W. Milne K. M. (2021). Pulmonary function and functional capacity in COVID-19 survivors with persistent dyspnoea. *Respir. Physiol. Neurobiol.* 288:103644. 10.1016/j.resp.2021.103644 33647535 PMC7910142

[B15] De Biasi S. Meschiari M. Gibellini L. Bellinazzi C. Borella R. Fidanza L. (2020). Marked T cell activation, senescence, exhaustion and skewing towards TH17 in patients with COVID-19 pneumonia. *Nat. Commun.* 11:3434. 10.1038/s41467-020-17292-4 32632085 PMC7338513

[B16] Del Turco S. Vianello A. Ragusa R. Caselli C. Basta G. (2020). COVID-19 and cardiovascular consequences: is the endothelial dysfunction the hardest challenge? *Thromb. Res.* 196 143–151. 10.1016/j.thromres.2020.08.039 32871306 PMC7451195

[B17] Dhont S. Derom E. van Braeckel E. Depuydt P. Lambrecht B. N. (2021). Conceptions of the pathophysiology of happy hypoxemia in COVID-19. *Respir. Res.* 22:12. 10.1186/s12931-021-01614-1 33419436 PMC7791161

[B18] Diao B. Wang C. Tan Y. Chen X. Liu Y. Y. Ning L. (2020). Reduction and functional exhaustion of T cells in patients with coronavirus disease 2019 (COVID-19). *Front. Immunol.* 11:827. 10.3389/fimmu.2020.00827 32425950 PMC7205903

[B19] Engelmann B. Massberg S. (2013). Thrombosis as an intravascular effector of innate immunity. *Nat. Rev. Immunol.* 13 34–45. 10.1038/nri3345 23222502

[B20] Frank K. Paust S. (2020). Dynamic natural killer cell and T cell responses to influenza infection. *Front. Cell. Infect. Microbiol.* 10:425. 10.3389/fcimb.2020.00425 32974217 PMC7461885

[B21] Frieman M. Heise M. Baric R. (2008). SARS coronavirus and innate immunity. *Virus Res.* 133 101–112. 10.1016/j.virusres.2007.03.015 17451827 PMC2292640

[B22] Frija-Masson J. Debray M.-P. Gilbert M. Lescure F.-X. Travert F. Borie R. (2020). Functional characteristics of patients with SARS-CoV-2 pneumonia at 30 days post-infection. *Eur. Respir. J.* 56:2001754. 10.1183/13993003.01754-2020 32554533 PMC7301832

[B23] Fumagalli A. Misuraca C. Bianchi A. Borsa N. Limonta S. Maggiolini S. (2020). Pulmonary function in patients surviving to COVID-19 pneumonia. *Infection* 49 153–157. 10.1007/s15010-020-01474-9 32725597 PMC7386387

[B24] Gordon D. E. Hiatt J. Bouhaddou M. Rezelj V. V. Ulferts S. Braberg H. (2020a). Comparative host-coronavirus protein interaction networks reveal pan-viral disease mechanisms. *Science* 370:eabe9403. 10.1126/science.abe9403 33060197 PMC7808408

[B25] Gordon D. E. Jang G. M. Bouhaddou M. Xu J. Obernier K. White K. M. (2020b). A SARS-CoV-2 protein interaction map reveals targets for drug repurposing. *Nature* 583 459–468. 10.1038/s41586-020-2286-9 32353859 PMC7431030

[B26] Graham B. L. Brusasco V. Burgos F. Cooper B. G. Jensen R. Kendrick A. (2017). 2017 ERS/ATS standards for single-breath carbon monoxide uptake in the lung. *Eur. Respir. J.* 49:1600016. 10.1183/13993003.00016-2016 28049168

[B27] Hikmet F. Méar L. Edvinsson Å Micke P. Uhlén M. Lindskog C. (2020). The protein expression profile of ACE2 in human tissues. *Mol. Syst. Biol.* 16:e9610. 10.15252/msb.20209610 32715618 PMC7383091

[B28] Hoffmann M. Kleine-Weber H. Schroeder S. Krüger N. Herrler T. Erichsen S. (2020). SARS-CoV-2 cell entry depends on ACE2 and TMPRSS2 and is blocked by a clinically proven protease inhibitor. *Cell* 181 271–280.e8. 10.1016/j.cell.2020.02.052 32142651 PMC7102627

[B29] Hou Y. J. Okuda K. Edwards C. E. Martinez D. R. Asakura T. Dinnon K. H. (2020). SARS-CoV-2 reverse genetics reveals a variable infection gradient in the respiratory tract. *Cell* 182 429–446.e14. 10.1016/j.cell.2020.05.042 32526206 PMC7250779

[B30] Hsieh M.-J. Lee W.-C. Cho H.-Y. Wu M.-F. Hu H.-C. Kao K.-C. (2018). Recovery of pulmonary functions, exercise capacity, and quality of life after pulmonary rehabilitation in survivors of ARDS due to severe influenza A (H1N1) pneumonitis. *Influenza Other Respir. Viruses* 12 643–648. 10.1111/irv.12566 29676537 PMC6086854

[B31] Huang Y. Tan C. Wu J. Chen M. Wang Z. Luo L. (2020). Impact of coronavirus disease 2019 on pulmonary function in early convalescence phase. *Respir. Res.* 21:163. 10.1186/s12931-020-01429-6 32600344 PMC7323373

[B32] Huertas A. Montani D. Savale L. Pichon J. Tu L. Parent F. (2020). Endothelial cell dysfunction: a major player in SARS-CoV-2 infection (COVID-19)? *Eur. Respir. J.* 56:2001634. 10.1183/13993003.01634-2020 32554538 PMC7301835

[B33] Hughes J. M. B. Pride N. B. (2012). Examination of the carbon monoxide diffusing capacity (D_L_*CO*__) in relation to its K_*CO*_ and V_*A*_ components. *Am. J. Respir. Crit. Care Med.* 186 132–139. 10.1164/rccm.201112-2160CI 22538804

[B34] Hui D. S. Joynt G. M. Wong K. T. Gomersall C. D. Li T. S. Antonio G. (2005a). Impact of severe acute respiratory syndrome (SARS) on pulmonary function, functional capacity and quality of life in a cohort of survivors. *Thorax* 60 401–409. 10.1136/thx.2004.030205 15860716 PMC1758905

[B35] Hui D. S. Wong K. T. Ko F. W. Tam L. S. Chan D. P. Woo J. (2005b). The 1-year impact of severe acute respiratory syndrome on pulmonary function, exercise capacity, and quality of life in a cohort of survivors. *Chest* 128 2247–2261. 10.1378/chest.128.4.2247 16236881 PMC7094276

[B36] Hui K. P. Y. Cheung M. C. Perera R. A. P. M. Ng K. C. Bui C. H. T. Ho J. C. W. (2020). Tropism, replication competence, and innate immune responses of the coronavirus SARS-CoV-2 in human respiratory tract and conjunctiva: an analysis in ex-vivo and in-vitro cultures. *Lancet Respir. Med.* 8 687–695. 10.1016/S2213-2600(20)30193-4 32386571 PMC7252187

[B37] Hulswit R. J. G. de Haan C. A. M. Bosch B.-J. (2016). Coronavirus spike protein and tropism changes. *Adv. Virus Res.* 96 29–57. 10.1016/bs.aivir.2016.08.004 27712627 PMC7112277

[B38] Iba T. Connors J. M. Levy J. H. (2020). The coagulopathy, endotheliopathy, and vasculitis of COVID-19. *Inflamm. Res.* 69 1181–1189. 10.1007/s00011-020-01401-6 32918567 PMC7486586

[B39] Jackson S. P. Darbousset R. Schoenwaelder S. M. (2019). Thromboinflammation: challenges of therapeutically targeting coagulation and other host defense mechanisms. *Blood* 133 906–918. 10.1182/blood-2018-11-882993 30642917

[B40] Jesenak M. Brndiarova M. Urbancikova I. Rennerova Z. Vojtkova J. Bobcakova A. (2020). Immune parameters and COVID-19 infection – associations with clinical severity and disease prognosis. *Front. Cell. Infect. Microbiol.* 10:364. 10.3389/fcimb.2020.00364 32695683 PMC7338601

[B41] Kopecky-Bromberg S. A. Martínez-Sobrido L. Frieman M. Baric R. A. Palese P. (2007). Severe acute respiratory syndrome coronavirus open reading frame (ORF) 3b, ORF 6, and nucleocapsid proteins function as interferon antagonists. *J. Virol.* 81 548–557. 10.1128/jvi.01782-06 17108024 PMC1797484

[B42] Kopf M. Schneider C. Nobs S. P. (2015). The development and function of lung-resident macrophages and dendritic cells. *Nat. Immunol.* 16 36–44. 10.1038/ni.3052 25521683

[B43] Kulikauskaite J. Wack A. (2020). Teaching old dogs new tricks? The plasticity of lung alveolar macrophage subsets. *Trends Immunol.* 41 864–877. 10.1016/j.it.2020.08.008 32896485 PMC7472979

[B44] Landsman L. Varol C. Jung S. (2007). Distinct differentiation potential of blood monocyte subsets in the lung. *J. Immunol.* 178 2000–2007. 10.4049/jimmunol.178.4.2000 17277103

[B45] Lazear H. M. Schoggins J. W. Diamond M. S. (2019). Shared and distinct functions of type I and type III interferons. *Immunity* 50 907–923. 10.1016/j.immuni.2019.03.025 30995506 PMC6839410

[B46] Lins M. Vandevenne J. Thillai M. Lavon B. R. Lanclus M. Bonte S. (2020). Assessment of small pulmonary blood vessels in COVID-19 patients using HRCT. *Acad. Radiol.* 27 1449–1455. 10.1016/j.acra.2020.07.019 32741657 PMC7381940

[B47] Liu K. Zhang W. Yang Y. Zhang J. Li Y. Chen Y. (2020). Respiratory rehabilitation in elderly patients with COVID-19: a randomized controlled study. *Complement. Ther. Clin. Pract.* 39:101166. 10.1016/j.ctcp.2020.101166 32379637 PMC7118596

[B48] Lukassen S. Chua R. L. Trefzer T. Kahn N. C. Schneider M. A. Muley T. (2020). SARS -CoV-2 receptor ACE 2 and TMPRSS 2 are primarily expressed in bronchial transient secretory cells. *EMBO J.* 39:e105114. 10.15252/embj.20105114 32246845 PMC7232010

[B49] Martin L. D. Rochelle L. G. Fischer B. M. Krunkosky T. M. Adler K. B. (1997). Airway epithelium as an effector of inflammation: molecular regulation of secondary mediators. *Eur. Respir. J.* 10 2139–2146. 10.1183/09031936.97.10092139 9311517

[B50] McGonagle D. O’Donnell J. S. Sharif K. Emery P. Bridgewood C. (2020). Immune mechanisms of pulmonary intravascular coagulopathy in COVID-19 pneumonia. *Lancet Rheumatol.* 2 e437–e445. 10.1016/S2665-9913(20)30121-1 32835247 PMC7252093

[B51] Meckiff B. J. Ramírez-Suástegui C. Fajardo V. Chee S. J. Kusnadi A. Simon H. (2020). Imbalance of regulatory and cytotoxic SARS-CoV-2-reactive CD4+ T cells in COVID-19. *Cell* 183 1340–1353.e16. 10.1016/j.cell.2020.10.001 33096020 PMC7534589

[B52] Mo X. Jian W. Su Z. Chen M. Peng H. Peng P. (2020). Abnormal pulmonary function in COVID-19 patients at time of hospital discharge. *Eur. Respir. J.* 55:2001217. 10.1183/13993003.01217-2020 32381497 PMC7236826

[B53] Morris M. F. Pershad Y. Kang P. Ridenour L. Lavon B. Lanclus M. (2021). Altered pulmonary blood volume distribution as a biomarker for predicting outcomes in COVID-19 disease. *Eur. Respir. J.* 2004133. 10.1183/13993003.04133-2020 33632795 PMC7908189

[B54] Ngai J. C. Ko F. W. Ng S. S. To K.-W. Tong M. Hui D. S. (2010). The long-term impact of severe acute respiratory syndrome on pulmonary function, exercise capacity and health status. *Respirology* 15 543–550. 10.1111/j.1440-1843.2010.01720.x 20337995 PMC7192220

[B55] Nusair S. (2020). Abnormal carbon monoxide diffusion capacity in COVID-19 patients at time of hospital discharge. *Eur. Respir. J.* 56:2001832. 10.1183/13993003.01832-2020 32703822 PMC7376285

[B56] Odak I. Barros-Martins J. Bošnjak B. Stahl K. David S. Wiesner O. (2020). Reappearance of effector T cells is associated with recovery from COVID-19. *EBioMedicine* 57:102885. 10.1016/j.ebiom.2020.102885 32650275 PMC7338277

[B57] Orme J. Romney J. S. Hopkins R. O. Pope D. Chan K. J. Thomsen G. (2003). Pulmonary function and health-related quality of life in survivors of acute respiratory distress syndrome. *Am. J. Respir. Crit. Care Med.* 167 690–694. 10.1164/rccm.200206-542OC 12493646

[B58] Park W. B. Jun K. I. Kim G. Choi J.-P. Rhee J.-Y. Cheon S. (2018). Correlation between pneumonia severity and pulmonary complications in Middle East respiratory syndrome. *J. Korean Med. Sci.* 33:e169. 10.3346/jkms.2018.33.e169 29892209 PMC5990444

[B59] Pellegrino R. (2005). Interpretative strategies for lung function tests. *Eur. Respir. J.* 26 948–968. 10.1183/09031936.05.00035205 16264058

[B60] Polak S. B. Van Gool I. C. Cohen D. von der Thüsen J. H. van Paassen J. (2020). A systematic review of pathological findings in COVID-19: a pathophysiological timeline and possible mechanisms of disease progression. *Mod. Pathol.* 33 2128–2138. 10.1038/s41379-020-0603-3 32572155 PMC7306927

[B61] Potus F. Mai V. Lebret M. Malenfant S. Breton-Gagnon E. Lajoie A. C. (2020). Novel insights on the pulmonary vascular consequences of COVID-19. *Am. J. Physiol. Lung Cell. Mol. Physiol.* 319 L277–L288. 10.1152/ajplung.00195.2020 32551862 PMC7414237

[B62] Qin W. Chen S. Zhang Y. Dong F. Zhang Z. Hu B. (2021). Diffusion capacity abnormalities for carbon monoxide in patients with COVID-19 at three-month follow-up. *Eur. Respir. J.* 2003677. 10.1183/13993003.03677-2020 33574077 PMC7877322

[B63] Rello J. Storti E. Belliato M. Serrano R. (2020). Clinical phenotypes of SARS-CoV-2: implications for clinicians and researchers. *Eur. Respir. J.* 55:2001028. 10.1183/13993003.01028-2020 32341111 PMC7236837

[B64] Rogliani P. Calzetta L. Coppola A. Puxeddu E. Sergiacomi G. D’Amato D. (2020). Are there pulmonary sequelae in patients recovering from COVID-19? *Respir. Res.* 21:286. 10.1186/s12931-020-01550-6 33126869 PMC7598236

[B65] Ruscitti P. Berardicurti O. Iagnocco A. Giacomelli R. (2020). Cytokine storm syndrome in severe COVID-19. *Autoimmun. Rev.* 19:102562. 10.1016/j.autrev.2020.102562 32376400 PMC7252135

[B66] Schmidt M. E. Varga S. M. (2018). The CD8 T cell response to respiratory virus infections. *Front. Immunol.* 9:678. 10.3389/fimmu.2018.00678 29686673 PMC5900024

[B67] Schultze J. L. Aschenbrenner A. C. (2021). COVID-19 and the human innate immune system. *Cell* 184 1671–1692. 10.1016/j.cell.2021.02.029 33743212 PMC7885626

[B68] Schurink B. Roos E. Radonic T. Barbe E. Bouman C. S. C. de Boer H. H. (2020). Viral presence and immunopathology in patients with lethal COVID-19: a prospective autopsy cohort study. *Lancet Microbe* 1 e290–e299. 10.1016/s2666-5247(20)30144-0 33015653 PMC7518879

[B69] Shi C. S. Nabar N. R. Huang N. N. Kehrl J. H. (2019). SARS-coronavirus open reading frame-8b triggers intracellular stress pathways and activates NLRP3 inflammasomes. *Cell Death Discov.* 5:101. 10.1038/s41420-019-0181-7 31231549 PMC6549181

[B70] Shi C.-S. Qi H.-Y. Boularan C. Huang N.-N. Abu-Asab M. Shelhamer J. H. (2014). SARS-coronavirus open reading frame-9b suppresses innate immunity by targeting mitochondria and the MAVS/TRAF3/TRAF6 signalosome. *J. Immunol.* 193 3080–3089. 10.4049/jimmunol.1303196 25135833 PMC4179872

[B71] Spagnolo P. Balestro E. Aliberti S. Cocconcelli E. Biondini D. Casa G. D. (2020). Pulmonary fibrosis secondary to COVID-19: a call to arms? *Lancet Respir. Med.* 8 750–752. 10.1016/S2213-2600(20)30222-8 32422177 PMC7228737

[B72] Sungnak W. Huang N. Bécavin C. Berg M. Queen R. Litvinukova M. (2020). SARS-CoV-2 entry factors are highly expressed in nasal epithelial cells together with innate immune genes. *Nat. Med.* 26 681–687. 10.1038/s41591-020-0868-6 32327758 PMC8637938

[B73] Tay M. Z. Poh C. M. Rénia L. MacAry P. A. Ng L. F. P. (2020). The trinity of COVID-19: immunity, inflammation and intervention. *Nat. Rev. Immunol.* 20 363–374. 10.1038/s41577-020-0311-8 32346093 PMC7187672

[B74] Thomas G. Tacke R. Hedrick C. C. Hanna R. N. (2015). Nonclassical patrolling monocyte function in the vasculature. *Arterioscler. Thromb. Vasc. Biol.* 35 1306–1316. 10.1161/ATVBAHA.114.304650 25838429 PMC4441550

[B75] Tian S. Hu W. Niu L. Liu H. Xu H. Xiao S.-Y. (2020a). Pulmonary pathology of early-phase 2019 novel coronavirus (COVID-19) pneumonia in two patients with lung cancer. *J. Thorac. Oncol.* 15 700–704. 10.1016/j.jtho.2020.02.010 32114094 PMC7128866

[B76] Tian S. Xiong Y. Liu H. Niu L. Guo J. Liao M. (2020b). Pathological study of the 2019 novel coronavirus disease (COVID-19) through postmortem core biopsies. *Mod. Pathol.* 33 1007–1014. 10.1038/s41379-020-0536-x 32291399 PMC7156231

[B77] Toufen C. Jr. Costa E. L. V. Hirota A. S. Li H. Y. Amato M. B. P. Carvalho C. R. R. (2011). Follow-up after acute respiratory distress syndrome caused by influenza a (H1N1) virus infection. *Clinics* 66 933–937. 10.1590/S1807-59322011000600002 21808854 PMC3129942

[B78] Varga Z. Flammer A. J. Steiger P. Haberecker M. Andermatt R. Zinkernagel A. S. (2020). Endothelial cell infection and endotheliitis in COVID-19. *Lancet* 395 1417–1418. 10.1016/S0140-6736(20)30937-5 32325026 PMC7172722

[B79] Vinayagam S. Sattu K. (2020). SARS-CoV-2 and coagulation disorders in different organs. *Life Sci.* 260:118431. 10.1016/j.lfs.2020.118431 32946915 PMC7490584

[B80] Wang Q. Zhang Y. Wu L. Niu S. Song C. Zhang Z. (2020). Structural and functional basis of SARS-CoV-2 entry by using human ACE2. *Cell* 181 894–904.e9. 10.1016/j.cell.2020.03.045 32275855 PMC7144619

[B81] Watanabe S. Alexander M. Misharin A. V. Budinger G. R. S. (2019). The role of macrophages in the resolution of inflammation. *J. Clin. Invest.* 129 2619–2628. 10.1172/JCI124615 31107246 PMC6597225

[B82] Way E. E. Chen K. Kolls J. K. (2013). Dysregulation in lung immunity - the protective and pathologic Th17 response in infection. *Eur. J. Immunol.* 43 3116–3124. 10.1002/eji.201343713 24130019 PMC3947216

[B83] Wu M. Chen Y. Xia H. Wang C. Tan C. Y. Cai X. (2020). Transcriptional and proteomic insights into the host response in fatal COVID-19 cases. *Proc. Natl. Acad. Sci. U.S.A.* 117 28336–28343. 10.1073/pnas.2018030117 33082228 PMC7668053

[B84] Wynn T. A. Vannella K. M. (2016). Macrophages in tissue repair, regeneration, and fibrosis. *Immunity* 44 450–462. 10.1016/j.immuni.2016.02.015 26982353 PMC4794754

[B85] Yoshikawa T. Hill T. Li K. Peters C. J. Tseng C.-T. K. (2009). Severe acute respiratory syndrome (SARS) coronavirus-induced lung epithelial cytokines exacerbate SARS pathogenesis by modulating intrinsic functions of monocyte-derived macrophages and dendritic cells. *J. Virol.* 83 3039–3048. 10.1128/JVI.01792-08 19004938 PMC2655569

[B86] Zhang M. Zhang S. (2020). T cells in fibrosis and fibrotic diseases. *Front. Immunol.* 11:1142. 10.3389/fimmu.2020.01142 32676074 PMC7333347

[B87] Zhang X. X. X. Tan Y. Ling Y. Lu G. Liu F. Yi Z. (2020). Viral and host factors related to the clinical outcome of COVID-19. *Nature* 583 437–440. 10.1038/s41586-020-2355-0 32434211

[B88] Zhao Y. M. Shang Y. M. Song W. B. Li Q. Q. Xie H. Xu Q. F. (2020). Follow-up study of the pulmonary function and related physiological characteristics of COVID-19 survivors three months after recovery. *EClinicalMedicine* 25:100463. 10.1016/j.eclinm.2020.100463 32838236 PMC7361108

[B89] Zhou R. To K. K. W. Wong Y. C. Liu L. Zhou B. Li X. (2020). Acute SARS-CoV-2 infection impairs dendritic cell and T cell responses. *Immunity* 53 864–877.e5. 10.1016/j.immuni.2020.07.026 32791036 PMC7402670

[B90] Ziegler C. G. K. Allon S. J. Nyquist S. K. Mbano I. M. Miao V. N. Tzouanas C. N. (2020). SARS-CoV-2 receptor ACE2 is an interferon-stimulated gene in human airway epithelial cells and is detected in specific cell subsets across tissues. *Cell* 181 1016–1035.e19. 10.1016/j.cell.2020.04.035 32413319 PMC7252096

[B91] Zuniga E. I. Macal M. Lewis G. M. Harker J. A. (2015). Innate and adaptive immune regulation during chronic viral infections. *Annu. Rev. Virol.* 2 573–597. 10.1146/annurev-virology-100114-055226 26958929 PMC4785831

